# IMCs Microstructure Evolution Dependence of Mechanical Properties for Ni/Sn/Ni Micro Solder-Joints

**DOI:** 10.3390/ma13010252

**Published:** 2020-01-06

**Authors:** Ning Ren, Heng Fang, Dong Wang, Chenyi Hou, Yatao Zhao, Fan Chen, Ye Tian, Kyung-Wook Paik, Yiping Wu

**Affiliations:** 1 School of Mechanical and Electrical Engineering, Henan University of Technology, Zhengzhou 450052, China; ningren001@126.com (N.R.); fhjl214@163.com (H.F.); wangdong23322@126.com (D.W.); houchenyi1@126.com (C.H.); zyt19970908@163.com (Y.Z.); 18638635780@163.com (F.C.); 2 School of Materials Science and Engineering, Huazhong University of Science and Technology, Wuhan 430074, China; ypwu@hust.edu.cn; 3 Department of Materials Science and Engineering, Korea Advanced Institute of Science and Technology, Daejeon 305-701, Korea

**Keywords:** micro-joints, intermetallic compounds, mechanical properties, failure mechanisms, reliability

## Abstract

The current miniaturization trend of microelectronic devices drives the size of solder joints to continually scale down. The miniaturized joints considerably increase intermetallic compounds (IMCs) volume fraction to trigger mechanical reliability issues. This study investigated precise relationships between varying IMC volumes and mechanical properties of Ni/Sn(20μm)/Ni micro-joints. A designed method that followed the IMC volume as the only variable was used to prepare micro-joint samples with different IMC volumes. The continuously thickened Ni_3_Sn_4_ IMCs exhibited a noticeable morphology evolution from rod-like to chunky shape. The subsequent tensile tests showed unexpected tensile strength responses as increasing Ni_3_Sn_4_ volume, which was strongly associated with the Ni_3_Sn_4_ morphological evolutions. Fractographic analysis displayed that the ductile fracture dominates the 20%–40% IMC micro-joints, whereas the brittle fracture governs the 40%–80% IMC micro-joints. For the ductile fracture-dominated joints, an abnormal reduction in strength occurred as increasing IMCs volume from 20% to 40%. This is primarily due to severe stress concentrations caused by the transformed long rod-typed morphology of the Ni_3_Sn_4_. For the brittle fracture-dominated joints, the strength appeared a monotonous increase as the Ni_3_Sn_4_ volume increased. This may be attributed to the increased crack resistance resulting from continuous coarsening of the chunky Ni_3_Sn_4_ without any voids. Moreover, the finite element analysis was provided to further understand the joint failure mechanisms.

## 1. Introduction

To date, micro solder-joints are regarded as critical components to realize vertical interconnections for chips stacking in three-dimensional (3D) through-silicon via (TSV) packaging [1,2]. With a sustained miniaturization for 3D TSV packaging, the micro solder-joints are being scaled down to be less than twenty microns from the current one hundred microns [3,4]. Such a dramatical reduction in size tends to result in the evolution of microstructure and mechanical properties of the micro-joints, and thereby triggering mechanical reliability issues [5,6]. The interfacial intermetallic compounds (IMCs) layer with a suitable thickness is an excellent indication of metallurgical bonding in solder joints [7]. However, due to totally different mechanical properties from the bulk solder, their excessive volume and growth may trigger mechanical property changes of solder joints [8]. Moreover, the morphology and phase stability of the IMCs were known to also affect the joints’ mechanical properties [9,10]. These effects will be severely identified as the IMCs volume increased in miniaturized solder joints. Therefore, it is of significance to investigate a precise dependence of IMCs growth on mechanical properties of micro solder-joints.

Micro solder-joints in 3D TSV packaging are commonly subjected to mechanical loading during serving [11]. It was well documented that the interfacial IMCs play an important role in the joint reliability because of their different mechanical properties [12,13]. However, the mechanical property effects dominated by higher volume fraction of the IMCs are not very well known. Currently, substantial literature is available to address impacts of IMC thickness on the joint mechanical properties. The varying thickness of the IMCs was commonly achieved by adjusting the reflowing temperature or isothermal aging time [14,15,16]. Hu et al. [14] studied the influences of IMC thickness on the tensile strength of Sn-based solder/Cu solder joints by changing reflow temperature. They observed that raising reflow temperature can induce a Cu_6_Sn_5_ thickness increase to cause an obvious reduction in the joint tensile strength. An et al. [15] investigated the influences of IMC thickness on the tensile strength and failure mode of Sn3.0Ag0.5Cu/Cu solder joints by isothermal aging. Their experiment results showed that prolonging aging time can increase the IMC thickness to result in a joint strength drop as well as a failure mode transition from the ductile fracture to the brittle fracture. It should be pointed out that these investigations ignore grains coarsening of the bulk solder that is extremely sensitive to the cooling rate of soldering and thermal aging. Such a grain coarsening phenomenon was reported to significantly weaken the joint mechanical property [16]. Furthermore, their experimental results generally showed that the bulk solder occupied much higher volume than the IMCs in the joint. As the IMCs dominated the miniaturized solder joints, the mechanical properties and failure mechanisms of these joints are anticipated to display a dramatic change. 

Additionally, most of the related investigations were primarily concentrated on the Cu/Sn-based solder/Cu interconnections. With the joints continually downsized, the Ni/Sn/Ni micro-joint should be favorable to be adopted as interconnection structure in 3D TSV packaging because of slow interfacial reactions and a unique reaction product (i.e., Ni_3_Sn_4_ intermetallic) [17,18]. More significantly, this intermetallic possesses better mechanical performance and higher fracture toughness compared to typical IMCs (i.e., Cu_6_Sn_5_ and Cu_3_Sn) [12,19,20]. However, to the author’s best knowledge, quite a few literature works investigated the influence of IMC volume variations on mechanical properties and failure mechanisms of Ni/solder/Ni solder joints, and especially no literature was found to really take the IMC effect alone into account.

Previous approaches to increase the IMC volume by raising the reflowing temperature or prolonging the aging time may be debatable since bulk solder grains tend to coarsen accompanied with the IMC growth. To emphasize IMC volume effects alone, these two phenomena must be decoupled in a designed method. A practicable method that extends the soldering time at a constant cooling rate enables the IMCs growth without changing bulk solder microstructural characteristics. This method was utilized in this study to prepare the Ni/Sn(20 μm)/Ni micro-joint samples with varying IMC volumes. The emphasis of this study was placed on a precise dependence of the IMCs volume on tensile properties and failure mechanisms of Ni/Sn/Ni micro-joints with a constant stand-off height of 20 μm using a tensile test.

## 2. Experimental and Numerical Procedures

### 2.1. Experimental Procedures

**Figure 1:**
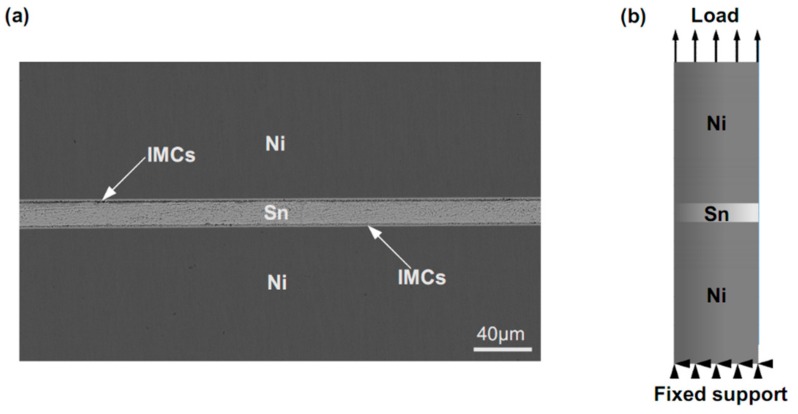
(**a**) cross-sectional SEM image of Ni/Sn/Ni samples after a standard reflow; (**b**) schematic diagram of constraints and loading conditions for a sample during tensile testing.

The experiment was designed to make the IMC volume as the only variable affect joint tensile responses. It was known that the factors such as varying stand-off heights of the joint, reflowing profile, and cooling rate commonly induced microstructure changes of the bulk solder and IMCs, leading to variations in joint tensile responses. Consequently, a reflow process was designed to fabricate micro-joint samples with varying IMC volumes by adjusting bonding times at a homogeneous temperature and a constant cooling rate. Simultaneously, a two-step method was designed to control a constant stand-off height of 20 μm for all the fabricated final samples. Firstly, a specially designed fixture was utilized to control the Sn interlayer height, and a stable solder reflow process at a maximum temperature of 250 °C for 40 s above the melting-point temperature of Sn was used to obtain the initial joint samples. The cross-sectional structure of the initial joint sample was provided in Figure 1, in which the IMC volume occupied about 10% in the joint. Based on the requirement for a constant stand-off height in the final samples with various IMCs volumes, the stand-off height in each initial sample is determined by the IMC volume contained in the final joint sample. Secondly, the initial Ni/Sn/Ni joint samples were fixed on the ceramic blocks to keep the initial joint stand-off height during the following reflow. A reflowing profile, with varying soldering times at a uniform soldering temperature of 265 °C and a constant cooling rate 2.5 °C/s, was employed to fabricate the joint samples with 20%, 40%, 60%, and 80% IMC volume fraction. All the samples were divided into two groups with each group containing the joint samples with different IMC volume fractions. The samples in one group were mounted in epoxy resin, and then ground and polished to characterize IMC microstructure evolutions. The tensile tests were carried out to examine tensile stress responses of the joints using the samples in another group. Backscattered electron (BSE) mode in a scanning electron microscope (SEM, Ultra-60, Zeiss, Oberkochen, Germany) was used to characterize microstructure evolutions of the micro-joints, and energy-dispersive X-ray spectrometry (EDS, 50mm-SDD, Oxford, Abingdon, UK) was utilized to examine the composition and elemental distribution of the IMCs. A common method was adopted to compute the IMC volume fraction in the micro-joint samples. In this method, the area fraction is assumed to represent the volume fraction, and a detailed description can be found in Ref. [21]. In the current study, ImageJ software (version, Manufacturer, City, US State abbrev. if applicable, Country) was used to measure the interfacial IMC area according to the cross-sectional image of the micro-solder joints.

### 2.2. Numerical Procedures

**Figure 2:**
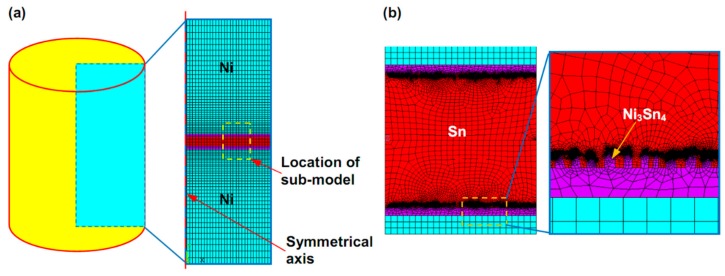
FE model of Ni/Sn/Ni micro-joints. (**a**) 2D axisymmetric global model; (**b**) sub-model containing rod-shaped IMCs.

**Figure 3:**
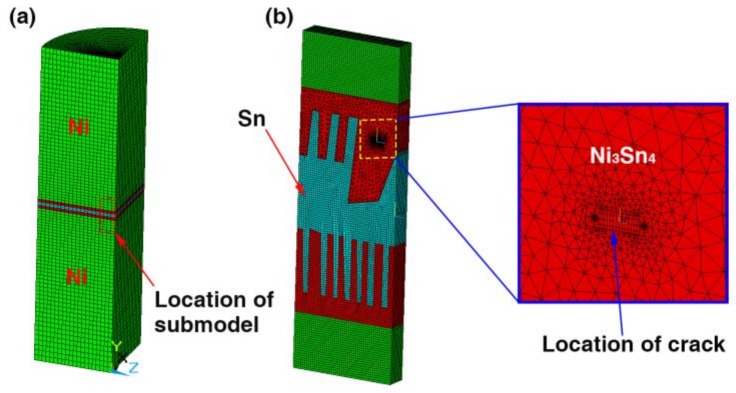
FE model of Ni/Sn/Ni micro-joints for (**a**) 3D 1/4 symmetric global model and (**b**) sub-model with a pre-existing crack at the chunky IMC surface.

Finite element (FE) method was implemented to deeply explain fracture mechanisms of the micro-joints subjected to tensile loading. Considering large difference in size between Sn interlayer and Ni/Sn/Ni structure as well as an axial symmetry of the joint, a method in combination of two-dimensional axisymmetric model with a sub-model containing the IMCs was developed according to SEM images of the 20%–40% IMCs joints in Figure 2. Furthermore, a 3D 1/4 symmetric model combined with a sub-model was established to understand impacts of the chunky IMC size on crack propagation resistance in the joints. A pre-existing semi-elliptical crack with a length of 0.5 µm was introduced into this sub-model as shown in Figure 3b. In the FE models, Ni and Ni_3_Sn_4_ were assumed to be linearly elastic, and Sn was taken to be linearly elastic up to a yield point. After the yield point, the solder followed plastic properties as presented in Table 1. The linearly elastic properties of the materials used in the FE analysis were listed in Table 2. Boundary constraints and tensile loading were applied depending on our experimental conditions, as seen in Figure 1b. 

## 3. Results and Discussion

### 3.1. Microstructure Characterization of the IMCs

**Figure 4:**
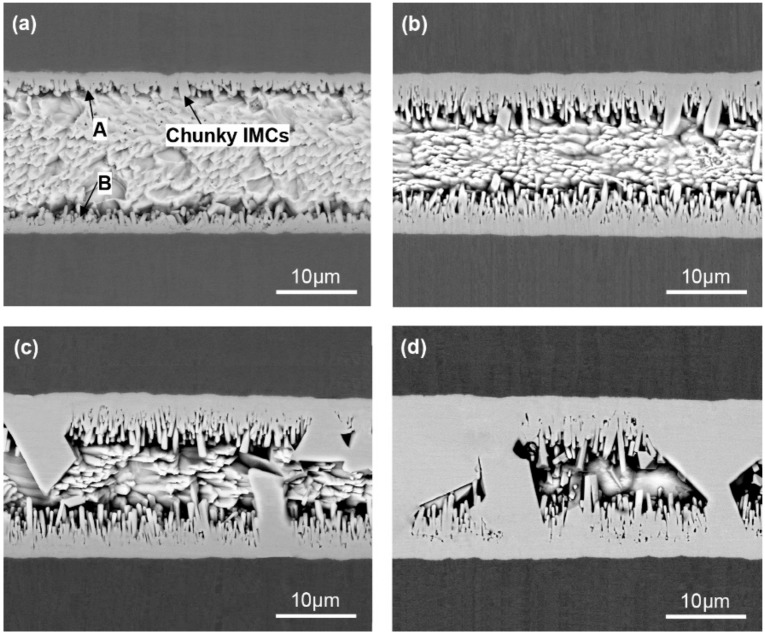
Cross-sectional SEM microstructure of Ni/Sn/Ni micro-joints as varying soldering times: (**a**) 7 min, (**b**) 21 min, (**c**) 50 min, and (**d**) 87 min.

Figure 4 shows the cross-sectional SEM images of Ni/Sn/Ni micro-joint samples with varying soldering times. At 7 min soldering time, a thin IMC layer with a short-rod shape was observed to form at the Ni/Sn interface from Figure 4a, and several small chunky IMCs also emerged at the interface. The IMC volume occupied around 20% in the joint. The EDS result in Table 3 combined with Ni-Sn binary phase diagram identified these IMCs as Ni_3_Sn_4_ intermetallic. At 21 min soldering time, the interfacial Ni_3_Sn_4_ grew obviously, and its volume fraction is approximately 40%. Moreover, the Ni_3_Sn_4_ morphology exhibited a remarkable change, i.e., the rod shape became significantly longer, the small chunky shape became larger. As the soldering time reached 50 min, the total interfacial Ni_3_Sn_4_ thickness continually increased, and the corresponding volume fraction increased up to 60%. It was worth noting that the contribution to the total Ni_3_Sn_4_ thickness increase is mainly attributed to the coarsened chunky Ni_3_Sn_4_ since the rod-shaped Ni_3_Sn_4_ thickness remains nearly unchanged. This particular phenomenon can be explained by the Ostwald Ripening theory. The large chunky Ni_3_Sn_4_ preferentially coarsened since the thickened Ni_3_Sn_4_ hindered the Ni atoms diffusion to cause no adequate Ni atoms for the rod-shaped Ni_3_Sn_4_ growth. When the soldering time increased up to 87 min as presented in Figure 4d, the continuously coarsened Ni_3_Sn_4_ intermetallic penetrated the whole joint cross-section to bridge the two opposite interfaces. The penetrating intermetallic was recently reported to have better electrical conductivity, thermal conductivity, and mechanical property [25]. Furthermore, a small number of voids were found to exist inside the rod-shaped IMCs. The formation of these voids is strongly related to the continuous growth of rod-shaped Ni_3_Sn_4_ IMCs, and the detailed explanation can be found in previous study [26].

### 3.2. IMC Growth Kinetics

Substantial researchers have investigated Ni_3_Sn_4_ growth kinetics during the reflowing process. An empirical power law was commonly used to describe IMC growth kinetics [27]:

(1) $$x=k\cdot t^{n},$$

where *x* is the IMC layer thickness at time *t*, *n* is the time exponent; *n* = 0.5 indicates that the reaction is controlled by bulk diffusion, and *n* = 0.33 indicates a reaction controlled by diffusion along grain boundaries. Equation (1) can be rewritten as:

(2) lnx=lnk+nlnt.

**Figure 5:**
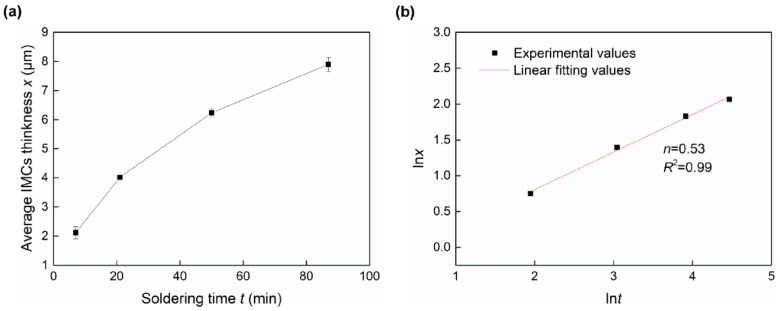
IMC layer thickness in one interfacial side vs. soldering time (**a**) *x*-*t* and (**b**) ln*x*-ln*t*.

In the current study, the calculated average Ni_3_Sn_4_ thickness in the joints with various soldering times was presented in Figure 5a. Using Equation (2), ln*x* versus ln*t* is plotted in Figure 5b to calculate time exponent (*n*). The value of *n* was determined by the slope of linear regression, as seen in Figure 5b. According to such a fitting, the kinetics parameter *n* was determined as 0.53. This value indicated that the bulk diffusion dominated the Ni_3_Sn_4_ growth at longer soldering times. Similar results were found in other studies [28,29]. Görlich et al. [28] reported that the Ni_3_Sn_4_ growth was controlled by grain boundary diffusion during the very early stages of the soldering, and the growth was transformed to be controlled by bulk diffusion at longer soldering times. Therefore, this growth mechanism is likely because the thickened Ni_3_Sn_4_ IMC layer hinders the Ni atoms diffusion to limit the interfacial reaction.

### 3.3. Influences of IMC Microstructure Evolution on Tensile Mechanical Properties of the Micro-Joints

**Figure 6:**
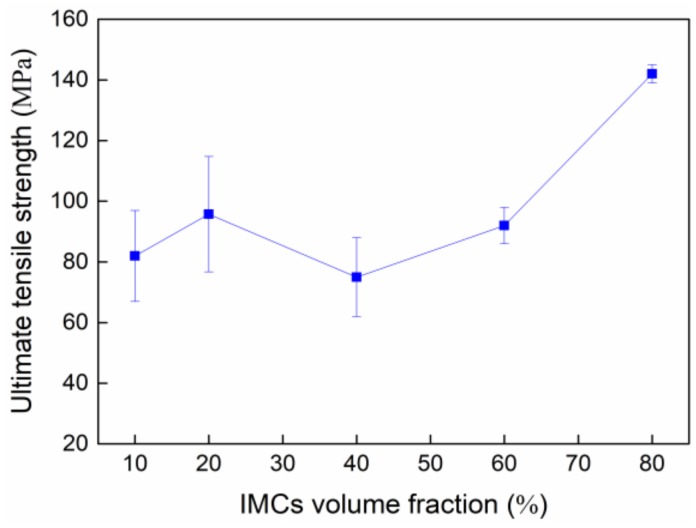
Ultimate tensile strength as a function of IMC volume fraction.

The tensile testing was performed to understand relationships of varying IMC volumes and joint mechanical properties; the tensile data were plotted in Figure 6. The ultimate tensile strength was observed to first increase to 96 MPa, and then drop to 75 MPa followed by a monotonous rise to a maximum value of 142 MPa as increasing IMC volume. Such a strength change is not agreed with other reports that showed a continuous drop in strength as increasing IMC volume. The detailed explanation with respect to this discrepancy would be given below. 

**Figure 7:**
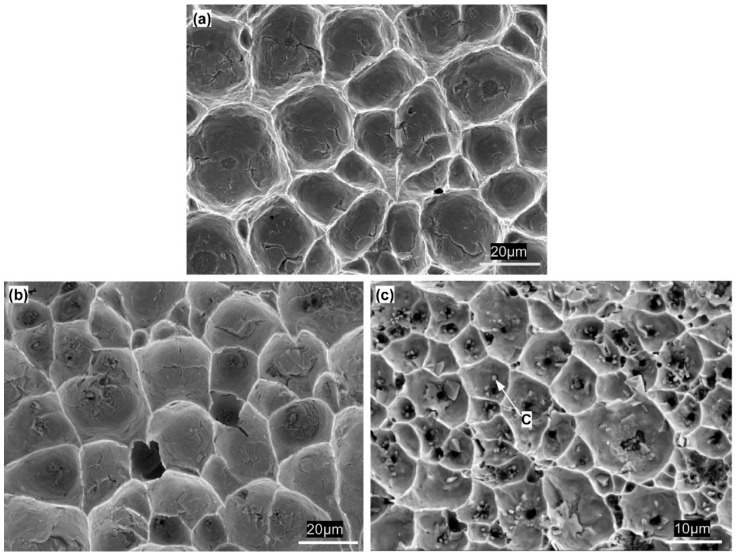
Fracture morphology of micro-joints with (**a**) 10%, (**b**) 20%, and (**c**) 40% IMC after tensile tests.

Figure 7 shows representative fracture surfaces of micro-joints with various IMC volumes after tensile testing. As presented in Figure 7a and b, the fracture surfaces were completely composed of equiaxed dimples in the 10% and 20% IMC micro-joints, indicating that the fracture mode is typical ductile fracture occurred into the bulk solder. Furthermore, the dimples size in the 10% IMC joint was visible to be larger than that in the 20% IMC joint; such a difference in dimples size is strongly related to the dimensional constraint. Clearly, the mechanical properties of these two joints are governed by the bulk solder. It was known that the joint mechanical properties dominated by the bulk solder primarily depends on the solder microstructure and dimensional constraint [20,30]. In the current study, the bulk solder microstructure in every sample nearly keeps identical as mentioned in Section 2. Therefore, the strength increase in the 20% IMC micro-joints is due to the enlarged dimensional constraint induced by the thinned solder layer, which can also rationalize the presence of smaller dimples in the 20% IMC micro-joints. 

**Figure 8:**
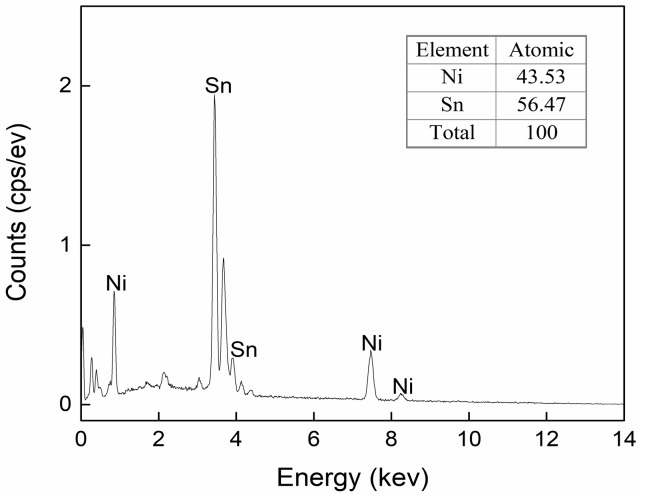
EDS analysis for the exposed IMCs at the bottom of dimples marked as C in Figure 7c.

**Figure 9:**
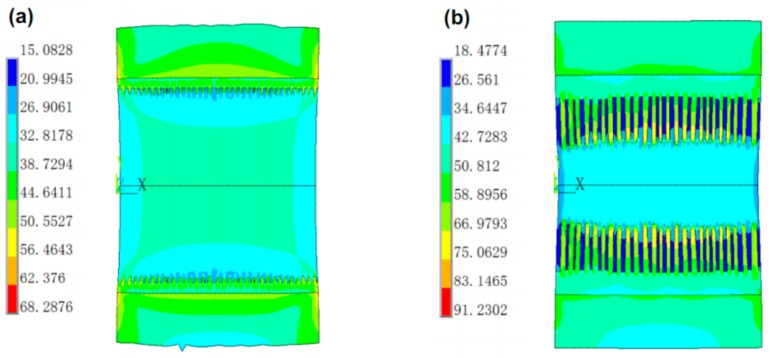
von-Mises stress (in MPa) contour plot for micro-joints with (**a**) 20% and (**b**) 40% IMCs under tensile loading. (For interpretation of the references to color in this figure legend, the reader was referred to the web version of this article.).

As shown in Figure 7c, as the IMCs volume increased to 40%, the dimples still occupied the whole fracture surface, but their size reduced significantly. Correlating the cross-sectional image of the interfacial IMCs in Figure 4b with Figure 7c, it is evident that many broken rod-shaped IMCs were exposed at the bottom of each of all the dimples. These exposed IMCs were identified as Ni_3_Sn_4_ intermetallic by EDS results in Figure 8. This suggested the fracture was located near the interface of the interfacial Ni_3_Sn_4_ and bulk solder. Furthermore, based on the previous discussion, the thinned solder layer can produce a larger dimensional constraint to strengthen the joint. However, the present study showed an unexpected tensile strength response that is a marked decrease in the 40% IMC joint. This abnormal response should be strongly associated with the Ni_3_Sn_4_ morphological transformation from short rod-like to long rod-like shape. The severe stress concentration may generate around the protruding tip of the long rod-shaped Ni_3_Sn_4_ to break them. This broken behavior facilitated the formation of substantial small broken dimples, and resulted in the strength reduction. As seen in Figure 7c, the exposed broken IMCs at the dimples bottom and the broken dimples can rationalize the above deduction. Figure 9 presents the von-Mises stress distribution in 20% and 40% IMC joints. The stress concentration was observed to emerge around the protruding tip of the long rod-shaped Ni_3_Sn_4_ in the 40% IMC joint, while the stress into the bulk solder is significantly higher than that near the solder/Ni_3_Sn4 interface in the 20% IMC joint. These simulative results can further verify the previous deduction for the strength reduction in the 40% IMC joints.

**Figure 10:**
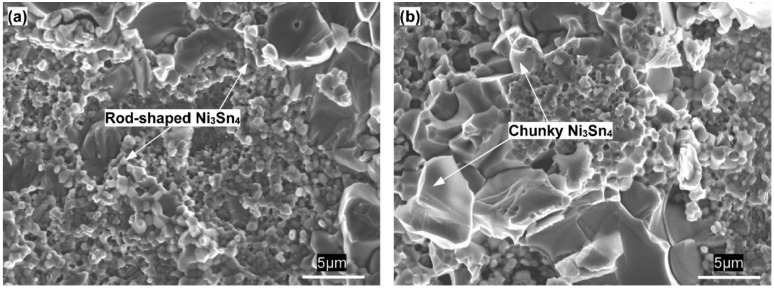
Fracture morphology of micro-joints with (**a**) 60% and (**b**) 80% IMCs after tensile tests.

Figure 10 shows the representative fracture surfaces of the 60% and 80% IMC micro-joints after tensile testing. The broken rod-shaped and chunky IMCs were visible to cover the whole fracture surface of these two joints, and the former dominates the 60% IMC joint while the latter is responsible for the 80% IMC joint. Apparently, these two failure modes belong to the brittle fracture mode happening in the IMCs. As seen in Figure 6, the ultimate tensile strength in the 40%–80% IMC joints displayed a significantly continuous increase. This result appears to be inconsistent with many previous investigations that demonstrated a strength reduction with increasing IMC volume [14,15]. Their explanation was mainly concentrated on the IMCs’ inherent brittleness. Actually, the IMCs were well known to possess considerably higher tensile strength than Sn-based solder materials, i.e., 2.0–5.1 GPa for Ni_3_Sn_4_ intermetallic and 86.1–96.7 MPa for Sn-rich solder [30,31]. Ho et al. [32] and Glenn et al. [33] reported that the presence of voids into the IMCs is a mainly influential factor in causing the joint rapid fracture due to low fracture toughness of IMCs. Kang et al. [34] and Tseng et al. [35] reported that a dual-layer IMC structure tends to cause premature joint failure. Consequently, the joint strength reduction with increasing IMC thickness should be attributed to the voids that existed in the IMCs as well as the dual-layer IMC structure. It is reasonable to suppose that the micro-joints composed of more volume fraction of single-phase IMCs without any voids may have higher tensile strength. In the current study, after 87 min soldering time, the chunky Ni_3_Sn_4_ intermetallic without any voids showed a rapid coarsening phenomenon, and even some large chunky Ni_3_Sn_4_ intermetallic coarsened to cross the whole joint as seen in Figure 4d. It should be noted that the Ni_3_Sn_4_ intermetallic is a unique reaction product between the molten Sn and solid-state Ni under 300 °C. As a result, the abnormal increase in tensile strength should be attributed to the coarsened single-phase Ni_3_Sn_4_ intermetallic without containing any voids. These IMCs may hinder the crack propagation to enhance the joint strength during the tensile process. In particular, the penetrating IMCs between the opposite interfaces fully dominate the joint strength due to higher strength and lower strain than Sn solder in the 80% IMCs joint; the corresponding joint strength was seen to reach the maximum value. 

The FE simulation was used to quantify the crack growth resistance of the chunky Ni_3_Sn_4_ with various volumes. According to linear elastic fracture mechanics, the crack propagation can be characterized by stress-intensity factors (SIFs, *K*_I_, *K*_II_ and *K*_III_). A lower SIFs means a higher crack growth resistance of the material. Given that the mode Ι (opening mode) fracture is inclined to happen in the solder joints subjected to the tensile loading [30], this work can assess the mode Ι SIFs using an interaction integral method. The interaction integral is expressed as [36]:

(3) $$I=-\int_{V}q_{i,j}\left[\sigma_{kl}\varepsilon_{kl}^{\mathrm{aux}}\delta_{ij}-\sigma_{kj}^{\mathrm{aux}}u_{k,i}-\sigma_{kj}u_{k,i}^{\mathrm{aux}}\right]dV/\int_{S}\delta q_{n}dS,$$

where *σ_ij_*, *ε_ij_*, and *u_k,i_* represent the stress, strain, and displacement components, respectively; $\sigma_{ij}^{\mathrm{aux}}$, $\varepsilon_{ij}^{\mathrm{aux}}$, and $u_{k,i}^{\mathrm{aux}}$ are the stress, strain, and displacement components of the auxiliary field, respectively; *δ_ij_* is the Kronecker delta, and *q_i_* is the crack-extension vector. The interaction integral is associated with the stress-intensity factors as [37]:

(4) $$I=2\left(1-\nu^{2}\right)K_{I}K_{I}^{\mathrm{aux}}/E,$$

where *ν* and *E* are the Poisson’s ratio and elastic modulus of Ni_3_Sn_4_, respectively; *K*_I_ is the mode I SIFs, and $K_{I}^{\mathrm{aux}}$ is the auxiliary mode I SIFs. The value of *K*_I_ in the chunky Ni_3_Sn_4_ can be obtained by the FE simulation. The maximum value of *K*_I_ in the larger and smaller chunky Ni_3_Sn_4_ is 1.1 MPa·mm^1/2^ and 1.5 MPa·mm^1/2^, respectively. It is apparent that increasing the chunky Ni_3_Sn_4_ size can enhance the crack growth resistance and thus the joint strength. This can validate the strength enhancement of the joint is primarily ascribed to the continuously coarsened chunky Ni_3_Sn_4_ in the 40%–80% IMC joints.

**Figure 11:**
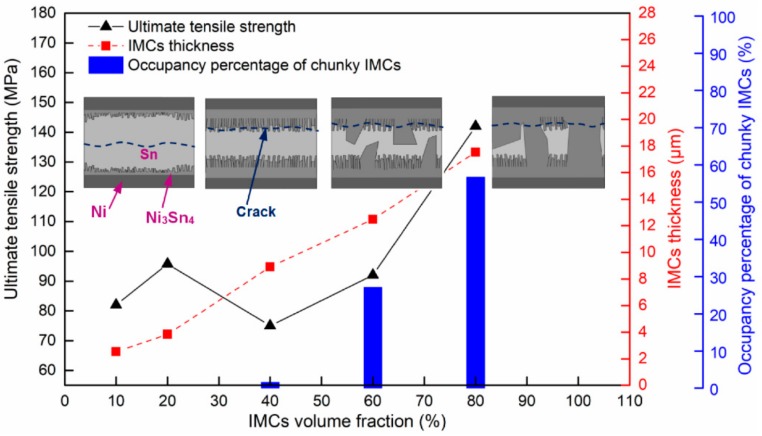
Relationships between the IMC microstructure and the micro-joints’ tensile mechanical properties, where schematic diagrams of the possible fracture path are also given.

Figure 11 summarizes the ultimate tensile strength and Ni_3_Sn_4_ morphologies as a function of Ni_3_Sn_4_ volume fraction, where schematic diagrams of the possible fracture paths in the micro-joints are also given. It is evident that the strength variations in the micro-joints are strongly linked to the IMC morphological evolutions. The transformed long rod-shaped Ni_3_Sn_4_ in the 40% IMC joint produced the severe stress concentration around their protruding tip, resulting in their fracture and the dimples rupture and thus the joint strength reduction. The fracture site transformed the interface of the Ni_3_Sn_4_ layer and the bulk solder from the bulk solder of the 10%–20% IMC joints. As the Ni_3_Sn_4_ volume increased, the thickened interfacial Ni_3_Sn_4_ significantly hindered the Ni atoms diffusion to limit the rod-shaped Ni_3_Sn_4_ growth but promoted the chunky Ni_3_Sn_4_ coarsening. The coarsened chunky Ni_3_Sn_4_ without containing any voids may improve the crack growth resistance during the tensile process, and thereby strengthen the joints. The corresponding fracture site transferred into the interfacial IMCs in the 60%–80% IMC joints, and thus the fracture mode converted to the brittle fracture from the ductile fracture in the 10%–40% IMC joints. Consequently, one concluded that mechanical properties of Ni/Sn/Ni micro-joints are primarily dependent on the volume and morphologies of the interfacial Ni_3_Sn_4_ intermetallic.

## 4. Conclusions

The precise dependence of IMC microstructure evolutions on tensile mechanical properties of Ni/Sn/Ni micro-joints with a constant stand-off height of 20 µm was investigated. The main findings can be summarized as follows:

(1) The Ni_3_Sn_4_ IMC volume increased obviously upon soldering time from 20% at 7 min to 80% at 87 min, and the Ni_3_Sn_4_ IMCs growth was determined to follow the bulk diffusion mechanism based on the kinetic parameter calculation. Simultaneously, the IMC morphology exhibited a noticeable evolution from rod-like to chunky shape.

(2) The unexpected joints’ strength occurrence is mainly due to the IMCs’ morphology evolutions. The transformed long rod-shaped Ni_3_Sn_4_ IMCs produced the severe stress concentration around their protruding tip, causing a joint strength drop. The following coarsened chunky IMCs without any voids may increase the crack growth resistance to enhance the joint strength.

(3) In 10%–40% IMC joints, the joints failure mode is the ductile fracture that occurred in the bulk solder, while the brittle fracture occurred into the Ni_3_Sn_4_ IMC layer in 60%–80% IMC joints. The dominated Ni_3_Sn_4_ IMCs morphology on the fracture surface switched to the chunky shape in 80% IMC joint from a rod-like shape in 60% IMC joint.

The present results can provide a better understanding of IMC microstructure evolution dependence of mechanical properties for Ni/Sn/Ni micro solder-joints. This can be beneficial to evaluate the mechanical reliability of Ni/Sn/Ni micro-joints and promote the utilization of Ni/Sn/Ni micro solder-joints in next generation 3D TSV packaging.

## Figures and Tables

**Table 1:** Plastic constants for Sn solder.

Material	Yield Strength (MPa)	Flow Region
Sn [22]	21.5	Stress *σ* = 37 × *ε*^0.075^, where *ε* is the strain from 0.001 to 0.016.

**Table 2:** Elastic material properties of the materials used in FE analysis.

Material	Elastic Modulus (MPa)	Poisson’s Ratio
Sn [22]	48	0.36
Ni [23]	207	0.312
Ni_3_Sn_4_ [24]	134	0.33

**Table 3:** EDS compositional analysis of IMC phases formed in Ni/Sn/Ni micro-joints.

Analysis Sites	Composition (at. %)		Phase
	Ni	Sn	
A	42.78	57.22	Ni_3_Sn_4_
B	42.99	57.01	Ni_3_Sn_4_
